# The ESG performance influence mechanism analysis-based on empirical analysis

**DOI:** 10.1371/journal.pone.0295548

**Published:** 2024-05-14

**Authors:** Lihua Ma, Xiuling Yuan, Jingyi Lu, Yifan Li, Weiqi Gao, Huizhe Yan, Xuedong Zhang

**Affiliations:** 1 School of Management Engineering and Business, Hebei University of Engineering, Handan, China; 2 University van Amsterdam, Amsterdam, Netherlands; 3 Department of Humanities Management, Seoul National University of Science and Technology, Nowon-gu, Seoul, Korea; University of Murcia: Universidad de Murcia, SPAIN

## Abstract

ESG has emerged as a prominent method for evaluating enterprises, gaining increasing importance in recent years. It assesses a company’s ability to promote sustainable economic development and fulfill its social responsibilities, encompassing three non-financial dimensions: environmental, social, and corporate governance. Regulatory authorities, industry associations, and investment institutions worldwide have placed growing emphasis on a company’s ESG performance. From the perspective of career concern, this study conducted a multiple regression analysis using data from Chinese A-share companies listed in Shanghai and Shenzhen from 2011 to 2020. It used CEO shareholding and CEO political affiliation as moderating variables to examine the impact of CEO career concerns on the corporate environment, society, and corporate governance performance. Empirical testing of whether CEO career concerns promote or suppress the ESG performance in enterprises. The findings of this study reveal that CEOs with heightened career concerns tend to impede the ESG performance of their respective enterprises. Additionally, CEO shareholding and political affiliations exert a negative moderating influence on the relationship between CEO career concerns and ESG performance. This research significantly extends the investigation into factors influencing ESG performance, offering fresh perspectives that could inform improved CEO oversight, foster corporate transformation, and enhance ESG performance.

## 1 Introduction

With the rapid development of China’s economy, environmental pollution issues are also constantly intensifying [1]. These issues encompass various concerns such as exhaust emissions from factory machinery, air pollution resulting from garbage incineration [2], and water pollution due to direct wastewater discharge from factories, among others [3]. The ramifications of environmental problems extend beyond personal health concerns and significantly constrain China’s economic development [4]. In the transition to a stage of high-quality economic development, the environment has emerged as a pivotal factor in China’s economic progress [5]. The economic development mode of most developing countries, including China, is mainly based on industrial development. Although this economic development mode primarily relies on a large amount of funds and resources to promote national economic development, it has caused severe environmental pollution. The concern of issue requires regulating the environment while developing the economy. Environmental regulation refers to the supervision of various behaviors that pollute the public environment intending to protect the environment [6]. With the increasing impact of the environment on the economy, the green finance industry has begun to flourish. Green finance is a financial service generated to coordinate economic growth and environmental protection, improve the efficiency of natural resource utilization, and is a tool for sustainable environmental development [7]. In the face of the non-systemic risks and profound uncertainties stemming from environmental change, ESG responsible investment, closely aligned with environmental protection, has gained prominence, evolving into a mainstream concept [8]. ESG is an emerging enterprise evaluation method that examines a company’s ability to respond to risks and long-term development from the perspectives of environmental, social performance, and corporate governance [9]. Enterprise ESG is mainly used in the investment field and has become an essential reference for influencing investment decisions. It evaluates the indicators of enterprises from three non-financial dimensions: environment, society, and corporate governance, and evaluates the contributions of enterprises (investment objects) in promoting sustainable economic development and fulfilling social responsibilities [10, 11]. The interplay between green finance and ESG is intricate and interconnected. The focus of green finance is on "green", which focuses more on the environmental aspect of ESG. In essence, ESG extends and enriches the principles of green finance [12].

The current literature research on the ESG system at home and abroad mainly focuses on three aspects: ESG information disclosure, ESG rating, and ESG investment [13]. ESG disclosures are the information basis for ratings and investments, ESG assessments provide evaluation and comparison methods, and ESG investments are based on the practice of both. The international academic community has accumulated rich theoretical achievements and practical experience in the ESG field. However, relevant research in China started relatively late, and in the field of ESG, there are still problems such as insufficient cognitive depth, lack of appropriate methodologies and indicator systems, inconsistent measurement standards, and a lack of samples from emerging capital markets [13]. There are two main perspectives on the career focus of CEOs for corporate decision-making. One view is that CEOs with high professional attention tend to use venture capital to obtain short-term returns and quickly demonstrate their abilities [14]. Another view suggests that CEOs with high professional attention tend to adopt more conservative or risk-averse methods to ensure future career continuity and performance stability [15]. This study focuses on analyzing whether CEO professional attention has an impact on corporate ESG performance.

This study analyzed a sample of Chinese companies listed on the Shanghai and Shenzhen A-share exchanges from 2010 to 2020 to empirically test the impact of CEO career concerns on the ESG performance of enterprises. The ESG data, CEO career focus data, company financial data, and corporate governance characteristics data involved in this study were sourced from the Bloomberg database, the China Securities Regulatory Commission database, the CSMAR database, and the CCER database. Most research on CEO career concerns focuses on micro-corporate behavior, without examining the impact of CEO career concerns on ESG performance from a macro perspective [16]. In addition, most existing studies only use a single ESG evaluation dimension as an explanatory variable, which lacks reliability, existing literature on the driving factors of corporate ESG performance focuses on the impact of financing constraints, corporate value, and internal governance on ESG decision-making [17], neglecting the influence of CEO personal traits.

In other to fill in the research gaps, this study analyzed a sample of Chinese companies to empirically test the impact of CEO career concerns on ESG performance of enterprises. The research purposes of this paper were as follows: (i) to explore the CEO’s professional attention effect on the ESG performance; (ii)to study the research on the impact of CEO shareholding and political background on CEO professional attention and corporate ESG performance, as well as the economic consequences of supplementing CEO professional attention.

This paper makes the following contributions to the study of CEO career focus and corporate ESG performance. The research contributions of this article are as follows: (i) to break away from the traditional perspective of studying the personal characteristics of executives and explores the impact of CEO personal characteristics on corporate decision-making based on upper level theory; (ii) to enriche and add literature on the economic consequences of CEO career concerns; (iii) to explore how CEO shareholding and political affiliation serve as moderating variables to influence the impact of CEO professional attention on corporate ESG performance.

## 2 Literature review and hypotheses

### 2.1 ESG performance and corporate sustainable development

In recent years, with a deepening understanding of sustainable development, local governments at all levels in China have allocated significant financial resources and implemented industrial policies to support industrial transformation and green development. As a result, the ESG (Environmental, Social, and Governance) performance of enterprises has seen substantial improvements. China has emerged as a major player in the global ESG market and has become the world’s second-largest green bond market. Research on ESG is also being carried out on a global scale. Tsang et al. [18] have summarized the literature on the determinants, characteristics, consequences, and moderators of ESG disclosure. Gillan et al. [19] reviewed the literature on the factors and economic consequences of ESG from the perspective of corporate finance. Naeem and Simin Chen et al. focused on the relationship between ESG and financial performance, while Li et al focused on the measurement and management of business performance [9, 15, 20]. The results showed that environmental, social, and governance performance had a substantial impact on firm behavior and outcomes, including firm market value, manager misconduct, and firm performance [21]. ESG demonstrated positive effects at both the macro and micro levels in the past, with the potential to increase firm market value and improve environmental and financial performance [22]. At the same time, it also could increase reputation and brand trust [24]. On the other hand, poor corporate ESG performance resulted in reputational or pollution-related costs, fines, and public warnings and criticism of corporate boards and managers in the past [19].

Based on the above research and analysis, the factors that have driven ESG performance in the past have been classified as external and internal. Concerning external factors, studies have shown that regulations, government actions and market forces have a significant impact on ESG performance. Cicchiello et al. found that China’s environmental protection tax improved environmental performance by encouraging green innovation, thereby enhancing firm ESG performance [23]. Li et al. found that the central environmental protection inspector improved the ESG performance of enterprises by strengthening the environmental compliance of enterprises, strengthening the connection between enterprises and stakeholders, and easing the agency conflicts between managers and shareholders [24]. Shu and TanIn the context of achieving the "two-carbon" goal, carbon control policy risk is negatively correlated with firm ESG performance, especially in non-state-owned enterprises, enterprises that are not sensitive to green innovation, carbon-sensitive industries, or regions with strict environmental regulations [25]. In addition to environmental regulations, government actions have also had an impact on ESG performance in the past. Nie et al. explored the crowding out effect of government debt on firm ESG performance. They found that government debt had crowded out credit resources, increased corporate financing costs, and reduced financial support for green governance [26]. This crowding out effect led to a significant decline in ESG performance. The weakening of government support for ESG also reduced enterprises’ enthusiasm for green innovation and willingness to assume social responsibility, which further negatively impacted enterprises’ ESG performance [27, 28].

In terms of internal factors, previous studies showed that digital finance, firm size, and green technology innovation were positively correlated with firm ESG performance [29–31]. Zhang et al. found that corporate social trust had a positive impact on ESG performance, and the presence of female directors in the senior management team enhanced corporate social trust [27]. Factors such as lower market competitiveness, a younger age of senior executives, and a larger scale of enterprises led to a more significant decline in ESG performance [32]. Kim et al., on the other hand, examined how a firm’s level of digitalization drove ESG performance [33]. However, they found that digital technology had not significantly affected companies’ environmental scores in the past. Similar findings were reported by Croci et al. and Cull et al. [34, 35]. On the government side, weakening environmental regulation and local governments’ urgent pursuit of economic growth weakened enterprises’ enthusiasm for green innovation and industrial upgrading in the past, which reduced green innovation ability and weakened ESG performance [36, 37]. Finally, the CEO has also been one of the key factors affecting ESG performance in the past. Some literature has suggested a strong correlation between a company’s ESG performance and the characteristics of its CEO and board. Research found that companies with female leaders or female board members had significantly higher ESG scores [38]. In addition, companies with younger CEOs were more likely to have better ESG performance [39]. Factors such as the CEO’s major, experience and gender also had some impact on the CEO’s career focus.

### 2.2 Relationship between CEO career concern and ESG performance

CEO concern refers to the personal and professional concerns and pressures that CEOs face during their tenure. Performance reviews were one of the factors that raised concerns about the CEO’s career. The results of a CEO’s performance review have a direct impact on their career development and compensation levels. Due to the limited information and capabilities of senior managers, they were not able to fully understand the company and its market environment. They could only rely on their cognition, experience, knowledge, and ability to make strategic decisions, which led to different organizational performance [40]. To achieve good evaluation results, the CEO is actively involved in the company’s strategic planning and financial and operational decisions.

Chen et al. pointed out that their professional focus decreased as their careers progressed, usually being higher early in their careers and lower at the end of their careers [41]. Thus, one view was that younger CEOs were more career-focused, more creative, and more willing to optimize resource allocation and improve work efficiency to achieve efficient business decisions. Managers typically took aggressive steps early in their careers to prove their competence, improve their early career performance for future income and career opportunities, and obtain better management market ratings. This meant that managers early in their careers had more significant career concerns and had a stronger desire to manage their income. Research found that younger CEOs were more likely to enter new business areas and exit existing ones. Younger CEOs were bolder in expanding and divesting, which can lead to significant increases and decreases in company size, respectively. Younger chief executives also preferred to grow through acquisitions rather than investing from scratch. However, this busy investment style of the young CEO didn’t seem to be hurting the efficiency of the company. At the same time, management turnover was influenced by firm performance and risk [42, 43]. CEOs with a high professional focus focused on the financial situation and performance management of their companies. They paid close attention to financial statements, fund flows, and profit growth and took the necessary steps to improve performance and reduce costs. Moreover, modern companies were increasingly focused on social and environmental impact, and young CEOs need to pay more attention to ESG investments. Cai et al. also highlighted the importance of a firm’s social reputation to a CEO’s career trajectory, especially in terms of corporate social responsibility [44]. They focused on corporate social image, corporate ethics and sustainability, and took relevant measures to promote corporate social responsibility and sustainable development. Based on the above studies, we propose the first hypothesis:

Hypothesis 1a: There is a significant positive correlation between CEO career focus and corporate ESG performance.

Another view was that managers tended to over-invest in ESG to achieve their own salary, career advancement, empire building, and political promotion. For the personal interests of managers, excessive ESG costs were borne by enterprises, resulting in an excessive concentration of enterprise resources on ESG projects. Ignoring investment needs in other key business areas limited overall ESG performance [45]. In addition, some studies have suggested that the high professional focus of CEOs may have led them to focus more on the short-term economic performance of their companies and personal financial returns, while ignoring ESG issues.

The perspective of the study found that short-term management ESG participation was reduced, which proved that giving priority to the short-term interests of managers hindered ESG activity participation [46]. Some CEOs also believed that focusing on ESG issues in a given situation increased the risks and costs faced by the company. As a result, they were reluctant to make investments and improvements in ESG, which inhibited ESG performance. In addition, CEOs with a high career focus lacked expertise and leadership on ESG issues. ESG is a complex field involving multiple dimensions and stakeholders. Without adequate expertise and skills, CEOs with a high career focus struggled to integrate and drive improvements in ESG performance effectively, and this lack of leadership hindered the organization’s continuous improvement and performance in ESG.

In the past, older CEOs, on the other hand, tended to be less career-focused and more focused on the long-term interests and sustainability of the company, with business decisions focused on integration, long-term orientation, risk management, innovation and research and development, and social responsibility and sustainability. Recent research had shown that older chief executives (CEOs) tended to be more competent, ethical, and risk-averse than their younger counterparts. Gillan et al. had also confirmed that corporate risk decreased with the age of the chief financial officer (CFO) [19]. Non-state-owned CEOs, older CEOs, and CEOs with lower shareholding ratios were more likely to have promoted corporate financialization later in their tenure. A large body of literature had also found that senior and older executives tended to be more competent than younger executives in everything from decision-making to improved company performance and better financial reporting [47–50]. Based on the above research, we propose a second hypothesis:

Hypothesis 1b: There is a significant negative correlation between CEO career focus and corporate ESG performance.

### 2.3 CEO other factors and corporate ESG performance

According to the theory of top management, the characteristics and experience of top management had an impact on corporate strategic decisions. The research primarily focused on the characteristics of the CEO or specific CEO situations in order to explain corporate social responsibility activities. For instance, these studies examined CEO pay structure, CEO career vision, CEO tenure, CEO hubbub and narcissism, CEO leadership style, and the political ideology of CEOs to explain the extent to which firms engaged in CSR [41, 51–58]. A large body of literature emphasized that CEO characteristics and preferences were important to company policies and outcomes [59]. It was well known that politically connected firms enjoyed competitive advantages, such as easier access to government investment, lower taxes, and more favorable regulatory conditions [60], and thus higher profits [59, 61]. Some studies further showed that the benefits of political relations on firm returns were more prominent in developing countries [61, 62], because political connections helped firms obtain low-cost investment projects and bank loans [62, 63]. Executive compensation was an important micro driving force of corporate strategy, and CEO ownership and risk factors also had an impact on executive behavior. Some studies had found that enterprises with social responsibility tended to have a relatively high equity compensation ratio and a relatively low cash compensation ratio [64]. Therefore, based on the existing studies, we proposed the following Hypothesis:

Hypothesis 1c: CEO other factors have a significant impact on corporate ESG performance.

## 3 Study design

ESG, as an emerging enterprise evaluation method in recent years, has a significant impact on financing costs and the sustainable development of enterprises. More and more people are researching research on ESG. Chen zhongfei et al used a sample of 3332 organizations worldwide from 2011 to 2020 for multiple regression analysis to analyze the impact of independent variables such as ESG rating on predictive variables and control variables such as corporate financial performance, to explore the impact of ESG on financial performance [65].

In the process of research design, through comprehensive consideration of other feasible analysis methods, other methods have certain drawbacks, such as: (i) Inability to perform multivariate analyses and effectively determine causality; (ii)Limited analytical capacity for actual data processing.

Therefore, we finally choose multiple regression analysis method to study the impact of CEO career attention on enterprise ESG performance. This choice is based on the following reasons: (i)Multiple regression analysis can simultaneously consider the influence of multiple independent variables on dependent variables, which is very important for the variables of multiple dimensions involved in our study; (ii) Multiple regression analysis provides the ability to control other possible influencing factors, which helps to ensure the accuracy and reliability of our research results; (iii)Multiple regression analysis method has been widely used in many empirical studies, and has the advantages of clear logic and strong interpretation, which enables us to comprehensively understand the impact mechanism of CEO career attention on enterprise ESG performance.

The importance of using multiple regression analysis in this study is reflected in the following aspects: (i)Multiple regression analysis can objectively evaluate the impact of CEO’s career attention, CEO’s shareholding situation and political relationship on enterprise ESG performance; (ii)Multiple regression analysis can quantify the strength of the relationship between CEO’s career attention, CEO’s shareholding and political relationship and enterprise ESG performance through correlation coefficient and regression coefficient. These quantitative metrics allow for a more accurate understanding of how each factor contributes to a company’s ESG performance; (iii)Control the influence of other variables: Multiple regression analysis can control other variables that may affect ESG performance of enterprises, so as to more accurately evaluate the independent influence of CEO’s career attention, CEO’s shareholding situation and political relationship on ESG performance of enterprises. (iv)Comprehensive consideration of multiple factors: Multiple regression analysis can simultaneously consider the impact of multiple influencing factors on enterprise ESG performance. By including CEO’s professional attention, CEO’s shareholding situation and political relationship into the model at the same time, the interaction and comprehensive effect between different factors can be revealed, so as to understand the formation mechanism of enterprise ESG performance more comprehensively; (v) The results of multiple regression analysis can provide scientific and quantitative policy suggestions for decision makers such as enterprise management, investors and regulators.

Using multiple regression analysis, the ESG performance of the firm is used as the dependent variable and the CEO career attention is used as the explanatory variable. By utilizing descriptive statistics, we can investigate potential variations in ESG performance across the sample companies based on diverse ESG indicators. Employing correlation analysis allows us to conduct an initial examination of the link between CEO career concerns and corporate ESG performance while also assessing the presence of collinearity issues. Through correlation analysis, it is possible to preliminarily explore the relationship between CEO career concerns and corporate ESG performance, and to determine whether there are collinearity issues. Through multivariate analysis, the relationship between CEO career attention and ESG performance can be further identified under the influence of different control variables. Heterogeneity tests were performed to examine how this relation varies among other factors. To test the validity and reliability of the results, robustness tests were used in this study, and potential endogeneity issues were discussed using instrumental variables and propensity score matching. Therefore, regression analysis is very appropriate. The degree and importance of correlations between variables can be assessed through regression analysis. The influence of the explanatory variable (CEO professional attention) on the dependent variable (ESG performance) can be determined by using regression to explain the influence of other variables (control variables).

### 3.1 Data

The ESG performance data consists of the ESG disclosure scores of the listed companies of Bloomberg and the ESG performance ratings of China Securities. The initial data for this study comes from Chinese A-share companies listed in the Shanghai and Shenzhen markets from 2011 to 2020. The acquisition of CEO personal characteristics in this study was obtained by organizing the personal information of executives and their resumes in the CSMAR database. We obtained CEO career key indicators from the annual reports of listed companies, company characteristics from the CSMAR database, and corporate governance characteristics from CCER. Considering distribution characteristics, data availability, and research samples, this study preprocessed the data by excluding ST, * ST, and companies in abnormal states; Integrate financial and insurance companies; Companies that have been listed for less than one year; And deleting company data that lacks variable data. This process resulted in the final sample of 28,754 annual observations. In order to control the impact of outliers, all continuous variables are winsorized at the top and bottom 1%.

### 3.2 Empirical section

#### 3.2.1 Dependent variable

The dependent variable is ESG performance. Overall indicators of ESG are receiving increasing attention due to investor demand, government regulation, and social pressures. At the end of the service, we compared ESG’s evaluation systems and found that they differ in terms of criteria, benchmarks, and coverage. We chose Bloomberg’s ESG rating and Huazheng’s ESG rating to measure the performance of listed companies. The Bloomberg ESG Disclosure Evaluation Index includes 22 weighted indicators to provide ESG ratings for companies, including environmental indicators such as air quality, climate change, water and energy management, materials and waste, and social indicators including ethics, human capital, health and safety. Corporate governance indicators include audit risk and supervision, board compensation, director diversity, director independence and structure, and shareholder governance indicators such as audit risk and supervision, director compensation, director diversification, director independence and structure, and shareholder tenure. The ESG indicator system is created based on industry characteristics and the Thomson Reuters importance matrix, where the industry weights the constructed distribution score matrix. This evaluation is based on publicly available information from investors, such as corporate social responsibility reports, annual reports, and websites, as well as a comprehensive evaluation of the three dimensions of ESG. Rating: 0.1 to 100. The China Securities ESG Indicator System is an indicator system based on the comprehensive framework of overseas ESG, taking into account the characteristics of China’s capital market and listed companies, such as poverty alleviation, corporate social responsibility, and secondary reporting sanctions. It is a three-layer indicator system, including 3 first level indicators, 14 second level indicators, and 26 third level indicators. This article adopts the industry weighted average method, combined with quarterly valuations and dynamics, and follows the ESG level calculations for listed companies. The ESG rating of China Securities is divided into nine levels in descending order: AAA, AA, A, BBB, BB, B, CCC, CC to C. We construct explanatory variables by dividing these ratings into 9–1 levels, with ESG = 9 when rated AAA, ESG = 1 when rated C, and so on.

#### 3.2.2 Independent variable

The explanatory variable is the professional concern of the CEO, as we focus on the impact of the CEO on changes in the company’s ESG. As Gillan and others explained, we chose the age of the CEO as an indicator of career focus. Due to the negative correlation between CEO age and career attention, we used the negative logarithm of CEO age to obtain attention for the main explanatory variable. Therefore, it more intuitively reflects the CEO’s attention on their career. The higher the value of attention, the higher the degree of the CEO’s attention to the profession.

#### 3.2.3 Control variables

Table 1 showed the variable definitions and measurements. Where t represents the year and i represents the company. ESG stands for ESG_ Bloomberg and ESG_ Indicator, which measures the performance of ESG for Huazheng Company. Concern represents the CEO’s professional attention. Year represents the year dummy variable, and it represents the industry dummy variable, α0 represents the intercept term, ε is a random perturbation term. α1 shows the degree to which the CEO’s professional focus has an impact on the company’s ESG performance. If α1 is negative, and the CEO’s professional concerns will have a negative impact on ESG performance; If α1 is positive, the comparison result is displayed.

**Table 1:** Variable definitions and measurements.

Variable type	Variable name	Variable Symbol	Variable definition
Dependent Variable	Bloomberg ESG	ESG_Bloomberg	Bloomberg ESG Evaluation Metrics
	China Securities ESG	ESG_Huazheng	CSI ESG Evaluation Index
Independent Variable	CEO Career Focus	Concern	Natural logarithm of the age of the CEO in the current period
Control Variables	Enterprise size	Size	Firm size measured by the natural logarithm of total assets
	Company Age	Age	Current year minus year of business registration
	Gearing ratio	Lev	Total liabilities at the end of a period/total assets at the end of the period
	Return on Net Assets	Roe	Net income/average shareholders’ equity
	Tobin’s Q value	TobinQ	(Market value of outstanding shares + market value of non-marketable shares + total long-term liabilities + total short-term liabilities at year-end)/total assets at year-end
	Shareholding Concentration	Top1	The proportion of shares held by the largest shareholder in the current year
	Percentage of independent directors	Indepen	Number of independent directors/Total number of board of directors
	Shareholding ratio of senior management	Ms	Number of shares held by management/total number of shares
	CEO/Chair duality	CEO dual	One person occupies the CEO and board chairperson positions equals 1; otherwise 0
	CEO education level	CEO edu	Master’s degree or above equals 1; otherwise 0
	CEO Tenure	CEO tenure	CEO term above the industry equals 1; otherwise 0
	CEO Overseas Experience	CEO overseas	CEO with overseas experience equals 1; otherwise 0
	CEO Gender	CEO gender	Women equal 1, men equal 0

### 3.3 Empirical model

To test the effect of CEO career focus (Concern) on ESG performance (ESG), we construct the following empirical model:

$$\begin{array}{cc} \text{ES}{\text{G}}_{\text{i},\text{t}} & =\alpha_{0}+\alpha_{1}\text{Concer}{\text{n}}_{\text{i},\text{t}-1}+\alpha_{2}\text{Concer}{\text{n}}_{\text{i},\text{t}-1}+\alpha_{3}\text{Siz}{\text{e}}_{\text{i},\text{t}-1}+\alpha_{4}\text{Ag}{\text{e}}_{\text{i},\text{t}-1}+\alpha_{5}\text{Le}{\text{v}}_{\text{i},\text{t}-1}\\ & +\alpha_{6}\text{Ro}{\text{e}}_{\text{i},\text{t}-1}+\alpha_{7}\text{Tobin}{\text{Q}}_{\text{i},\text{t}-1}+\alpha_{8}\text{Top}1_{\text{i},\text{t}-1}+\alpha_{9}\text{Indepe}{\text{n}}_{\text{i},\text{t}-1}\\ & +\alpha_{9}\text{Indepe}{\text{n}}_{\text{i},\text{t}-1}+\alpha_{9}\text{Indepe}{\text{n}}_{\text{i},\text{t}-1}+\alpha_{10}\text{M}{\text{s}}_{\text{i},\text{t}-1}+\alpha_{11}\text{CEO}\;\text{dua}{\text{l}}_{\text{i},\text{t}-1}\\ & +\alpha_{12}\text{CEO}\;\text{ed}{\text{u}}_{\text{i},\text{t}-1}+\alpha_{13}\text{CEO}\;\text{tenur}{\text{e}}_{\text{i},\text{t}-1}+\alpha_{14}\text{CEO}\;\text{oversea}{\text{s}}_{\text{i},\text{t}-1}\\ & +\alpha_{15}\text{CEO}\;\text{gende}{\text{r}}_{\text{i},\text{t}-1}+\sum \text{Year}+\sum \text{Int}+\varepsilon_{\text{i},\text{t}}, \end{array}$$ (1)

where i and t represent the firm and year, respectively, ESG represents the indicators ESG Bloomberg and ESG Huazheng, which measure the firm’s ESG performance, and Concern represents the CEO career concern variable. Year represents the year dummy variable, Int represents the industry dummy variable, α0 represents the intercept term, and ε is the random disturbance term. The coefficient α1 represents the degree of influence of CEO career concern on corporate ESG performance. If α1 is negative, then CEO career concern has a negative influence on ESG performance; if α1 is positive, it shows contrasting results.

## 4 Empirical results

### 4.1 Descriptive statistics

Table 2 showed the descriptive statistics. The total sample quantity for ESG Bloomberg was 9,726, the average value for ESG Bloomberg was 19.23, and the standard value, minimal value, maximal value, and median value were 6.802, 8.045, 52.13 and 18.42, respectively. The total sample size of ESG Huazheng is 28,754, the mean value is 6.012, and the standard deviation, minimum, maximum and mean values are1.046, 1, 8.526 and 6, respectively. CEO Concern has a total sample size of 29,012 with a mean of -3.415. The standard deviation, minimum value, maximum value and mean value are 0.191, -4.012, -3.546 and 3.856, respectively. The total sample Size of enterprise size (Size) is 29,012, and the mean value is 20.23. The standard deviation, minimum, maximum and average values are 1.415, 18.16, 27.12 and 20.142 respectively. The total sample size of enterprise Age (Age) is 29,012, the mean value is 19.46, and the standard deviation, minimum, maximum and mean value are 5.935, 1.625, 32.462 and 16.225, respectively. The total sample size of the enterprise asset-liability ratio (Lev) was 29,012, the mean value was 0.481, and the standard deviation, minimum, maximum and mean values were 0.745, 0.0635, 0.985 and 0.965, respectively. The total sample size of the average return on equity (Roe) of enterprises is 29,012, the average is 0.0562, and the standard deviation, minimum, maximum and average are 0.362, -2.488, 0.446 and 0.0748, respectively. The total sample size of enterprise ownership concentration (Top1) is 29,012, the average value is 0.362, and the standard deviation, minimum, maximum and average values are 0.265, 0.0823, 0.956 and 0.395, respectively. The total sample size of independent directors (Indepen) is 29,012, with a mean of 0.416 and a standard deviation of 0.194. The total sample size of executive stock ownership (MS) is 29,012, with a mean of 0.0612 and a standard deviation of 0.102. The total sample size for CEO dual is 29,012, with a mean of 0.956 and a standard deviation of 0.496. The total sample size for whether the CEO and a graduate degree (CEO edu) was 29,012, with a mean of 0.0581and a standard deviation of 0.475. The total sample size for CEO tenure is 29,012, with a mean of 0.532 and a standard deviation of 0.543. The total sample size for CEO overseas experience was 29,012, with a mean of 0.0596 and a standard deviation of 0.266. The total sample size for CEO gender was 29,012, with a mean of 0.0965 and a standard deviation of 0.146.

**Table 2:** Descriptive statistics.

Variable	N	mean	sd	min	p50	max
ESG_Bloomberg	9726	19.23	6.802	8.045	18.42	52.13
ESG_Huazheng	28754	6.012	1.046	1	6	8.526
Concern	29012	-3.415	0.191	-4.012	-3.856	-3.546
Size	29012	20.23	1.415	18.16	20.142	27.12
Age	29012	19.46	5.935	1.625	16.225	32.462
Lev	29012	0.481	0.745	0.0635	0.965	0.985
Roe	29012	0.0562	0.362	-2.488	0.0748	0.446
TobinQ	29012	2.849	2.126	0.785	2.036	20.32
Top1	29012	0.362	0.265	0.0823	0.395	0.956
Indepen	29012	0.416	0.194	0	0.346	0.984
Ms	29012	0.0612	0.102	0	0.000326	0.512
CEO dual	29012	0.956	0.496	0	0	1
CEO edu	29012	0.581	0.475	0	1	1
CEO tenure	29012	0.532	0.543	0	0	1
CEO overseas	29012	0.0596	0.266	0	0	1
CEO gender	29012	0.0965	0.146	0	0	1

### 4.2 Correlation analysis

Table 3 presents the Pearson correlation coefficients of the variables. Among them, the correlation coefficient between ESG Bloomberg and ESG Huazheng is 0.316***. This indicates that there is a positive and significant linear correlation between them. It follows that these two variables are affected by the same factors. The correlation coefficient between ESG Bloomberg and CEO occupational focus is -0.182***, indicating that there is a negative and significant linear correlation between them. The correlation coefficient between ESG Huazheng and CEO occupational attention is -0.062***, indicating that ESG Huazheng and CEO occupational attention are also significantly negatively correlated. Based on the above data, we tentatively believe that there is a significant negative correlation between CEO career focus and enterprise ESG performance. Among other dimensions that affect CEO career concerns, whether the dual role of chairman and CEO has a significant negative correlation with corporate ESG performance, CEO education has a significant positive correlation with corporate ESG performance, and CEO tenure has a significant positive correlation with corporate CEO performance. At the 5% significance level, the CEO’s overseas experience was positively correlated with ESG Bloomberg, while CEO gender was negatively correlated with ESG Bloomberg. These data validate the hypothesis that there is a significant negative correlation between CEO career focus and enterprise ESG performance. Finally, we build a multiple regression model and find that the absolute values of the correlation coefficients between each variable are less than 0.5, indicating that there is no serious multicollinearity problem and that the conclusions are relatively accurate.

**Table 3:** Correlation matrix.

	ESG_Bloomberg	ESG_Huazheng	Concern	Size	Age	Lev	Roe	TobinQ	Top1	Indepen	Ms	CEO dual	CEO edu	CEO te~e	CEO overseas	CEO gender
ESG_Bloomberg	1															
ESG_Huazheng	0.316***	1														
Concern	-0.182**	-0.062***	1													
Size	0.536***	0.485***	-0.123***	1												
Age	0.245***	0.063***	-0.103***	0.163***	1											
Lev	0.192***	0.092***	-0.026***	0.512***	0.126***	1										
Roe	0.067***	0.185***	-0.073***	0.095***	-0.065***	-0.185***	1									
TobinQ	-0.336***	-0.104***	0.032***	-0.546***	-0.123***	-0.313***	0.052***	1								
Top1	0.081***	0.163***	-0.052***	0.295***	-0.195***	0.056***	0.195***	-0.095***	1							
Indepen	-0.043***	0.057***	0.037***	-0.075***	-0.102***	-0.034***	0.056***	0.071***	0.056***	1						
Ms	-0.195***	-0.095***	-0.025**	-0.213***	-0.237***	-0.416***	0.063***	0.223***	-0.065***	0.078***	1					
CEO dual	-0.185***	-0.175***	-0.199***	-0.285***	-0.165***	-0.395***	0	0.193***	-0.043***	0.004***	0.485***	1				
CEO edu	0.081***	0.062***	0.055***	0.166***	0.123***	0.116***	-0.024***	-0.046***	0.095***	-0.031*	-0.086***	-0.023***	1			
CEO tenure	0.032**	0.034***	-0.150***	0.045***	0.068***	-0.048**	0.036**	-0.013***	-0.061***	0.095***	0.034***	0.095***	-0.089***	1		
CEO overseas	0.019***	-0.004**	0.095***	-0.007**	-0.062***	-0.062***	0.042**	0.095***	-0.032***	0.004**	0.032***	0.031***	0.063***	0.036**	1	
CEO gender	-0.043***	-0.006***	0.046***	-0.036***	0.034***	-0.062***	0.026*	0.081***	0.005**	0.034***	0.019***	-0.026***	-0.016**	0.00300	0.017**	1

*, **, and *** denote statistical significance at the 10, 5, and 1% levels, respectively. t-values are in parentheses.

### 4.3 Multivariate analysis

Table 4 lists the regression results of Tobit model for each variable. Model 1 shows a negative correlation between ESG Bloomberg and CEO occupational concern (α = -0.602, β = -1.13,), that is, a higher ESG Bloomberg score is generally associated with a lower concern number. A lower ESG Bloomberg score is generally associated with a higher concern number. After adding CEO’s personal characteristics as control variables in model 3, α = -1.196***, β = -2.85, indicating that the negative correlation between ESG Bloomberg and concern is more significant when the influence of CEO’s personal characteristics is taken into account. Similarly, Model 2 and Model 4 show a significant negative correlation between concern and ESG response at the 1% level. This suggests that the occupational focus of CEOs inhibits firm ESG performance, and the results support hypothesis 1b.

**Table 4:** CEO career focus and ESG performance.

	(1)	(2)	(3)	(4)
	ESG_Bloomberg	ESG_Huazheng	ESG_Bloomberg	ESG_Huazheng
Concern	-0.602	-0.263***	-1.196***	-0.384**
	(-1.13)	(-7.23)	(-2.85)	(-8.13)
Size	2.195**	0.395***	2.195**	0.395**
	(32.34)	(59.13)	(30.13)	(55.13)
Age	0.0956*	0.005***	0.084***	0.005**
	(7.42)	(5.63)	(7.85)	(4.98)
Lev	-2.598**	-0.985***	-2.695***	-0.748***
	(-6.95)	(-23.51)	(-6.63)	(-18.62)
Roe	0.712*	0.556**	0.846**	0.545***
	(2.66)	(12.36)	(2.02)	(15.11)
TobinQ	-0.195**	0.092***	-0.196***	0.056***
	(-2.84)	(7.61)	(-2.84)	(7.95)
Top1	1.203**	0.293***	1.245**	0.469***
	(2.95)	(8.13)	(2.88)	(7.36)
Indepen	2.155**	0.199***	2.023***	0.295***
	(3.12)	(3.12)	(3.88)	(2.963)
Ms	-1.885***	-0.096**	-0.419	0.073**
	(-2.66)	(-1.86)	(-0.69)	(2.013)
CEO_dual			-0.856***	-0.195***
			(-6.84)	(-8.96)
CEO_edu			0.563***	0.034***
			(3.85)	(6.82)
CEO_tenure			0.295*	0.063***
			(3.416)	(5.46)
CEO_overseas			0.695**	0.022
			(2.96)	(0.91)
CEO_gender			-0.322	-0.023
			(-1.41)	(-0.65)
_cons	-33.623**	-3.003**	-36.859***	-3.956***
	(-12.34)	(-13.92)	(-15.26)	(-12.36)
Year	Yes	Yes	Yes	Yes
Ind	Yes	Yes	Yes	Yes
N	9726	29012	9702	29012
adj. R2	0.362	0.246	0.362	0.222

*, **, and *** denote statistical significance at the 10, 5, and 1% levels, respectively. t-values are in parentheses.

Table 4 shows the influence of enterprise factors on enterprise ESG performance. Firm Size and ESG performance are significantly positively correlated at the 1% level. Firm age (age) has a significant positive correlation with ESG performance at the 1% level. Asset-liability ratio (Lev) and ESG performance are significantly negatively correlated at 1%. Return on equity (Roe) is significantly positively correlated with ESG performance at the 5% and 1% levels. TobinQ was significantly negatively correlated with ESG performance at the 1% level. Stock concentration (Top1) is significantly positively correlated with ESG performance at the 1% level. The percentage of independent directors (Indepen) is significantly positively correlated with ESG performance at the 1% level. Executive ownership (Ms) is negatively correlated with overall ESG performance.

Table 4 also shows the influence of various factors of the CEO on the ESG performance of enterprises. There is a significant negative correlation between the dual role of chairman and CEO and ESG performance at a 1% significance level. The level of education (CEO edu) was positively correlated with ESG performance at the 1% significance level. CEO tenure is positively correlated with ESG performance at the significance levels of 5% and 1%. There is a significant positive correlation between overseas CEOs and ESG performance at a significance level of 5%. There was no significant correlation between gender and ESG performance.

### 4.4 Robustness tests

#### 4.4.1 Propensity Score Matching (PSM)

We divided the sample data into two groups based on the median age of CEOs: young CEOs under 49 years old in the treatment group and older CEOs over 49 years old in the control group. Then we select a set of characteristic variables that will affect CEO selection behavior, including CEO’s age, education level, overseas experience, etc, and introduce control variables into the basic regression equation. We then use these characteristic and control variables to estimate the probability that the CEO chooses a company with short-term performance to obtain a propensity score. Subsequently, we paired each CEO from the experimental group with a CEO from the control group using a one-to-one non-relaxed nearest neighbor approach. The goal of the matching was to ensure that the CEOs in the trial and control groups were as similar in their propensity scores as possible, and that the difference in propensity scores between the experimental and control groups did not exceed 5%. In the end, the results are shown in columns 1 and 2 of Table 5. The results show that the negative correlation between CEO career focus and ESG performance remains significant in the matched samples, indicating that the analysis results are robust.

**Table 5:** Robustness tests.

	PSM		IV		Future Phase IY	
	ESG_Bloomberg	ESG_Huazheng	ESG_Bloomberg	ESG_Huazheng	ESG_Bloomberg	ESG_Huazheng
Concern	-1.856***	-0.656***	-3.926**	-0.882**	-0.903**	-0.344***
	(-4.31)	(-9.45)	(-1.84)	(-1.93)	(-1.95)	(-7.85)
Size	2.026***	0.385***	1.684***	0.285***	2.185***	0.385***
	(32.49)	(51.26)	(12.102)	(14.62)	(32.16)	(50.49)
Age	0.198***	0.002	0.689	-0.052	0.095***	0.008***
	(9.84)	(0.48)	(1.84)	(-0.64)	(6.75)	(6.35)
Lev	-2.362***	-0.635***	-1.224***	-0.688***	-2.685***	-0.587***
	(-4.19)	(-17.21)	(-3.71)	(-14.62)	(-5.13)	(-15.21)
Roe	0.632***	0.588***	0.195	0.284***	1.956***	0.885***
	(3.75)	(12.63)	(0.93)	(10.45)	(4.77)	(16.49)
TobinQ	-0.062	0.082***	0.193***	0.008*	-0.156***	0.043***
	(-0.94)	(6.95)	(4.74)	(1.92)	(-2.95)	(7.86)
Top1	0.712*	0.416***	2.152***	0.488***	0.812**	0.312***
	(1.92)	(6.92)	(3.35)	(6.82)	(2.63)	(6.46)
Indepen	1.145**	0.496**	1.462**	0.062	2.295***	0.1883***
	(2.49)	(2.66)	(2.95)	(1.49)	(3.73)	(2.94)
Ms	0.578	0.192**	0.956	0.481***	0.495	-0.063
	(0.46)	(2.46)	(1.84)	(7.75)	(0.632)	(-0.69)
CEO_dual	-1.295***	-0.193***	-0.684**	-0.196***	-0.849***	-0.106***
	(-8.41)	(-10.24)	(-2.63)	(-4.45)	(-5.16)	(-7.46)
CEO_edu	0.295	0.046***	0.195	0.034*	0.895***	0.083***
	(1.846)	(6.92)	(1.85)	(1.95)	(3.75)	(5.95)
CEO_tenure	0.574***	0.196***	-0.369	0.013	0.069	0.041**
	(5.95)	(8.74)	(-1.45)	(0.67)	(0.46)	(3.49)
CEO_overseas	0.036	0.056	-0.296	0.022	0.956***	0.056
	(0.08)	(1.95)	(-1.74)	(1.81)	(2.874)	(1.09)
CEO_gender	-0.152	0.035	0.845***	-0.063	-0.362	-0.035
	(-1.95)	(1.75)	(2.96)	(-1.81)	(-1.914)	(-0.64)
_cons	-40.242***	-2.845***	-40.342***	-0.958	-38.162***	-3.695***
	(-14.37)	(-10.36)	(-2.916)	(-0.67)	(-12.345)	(-12.32)
Year	Yes	Yes	Yes	Yes	Yes	Yes
Ind	Yes	Yes	Yes	Yes	Yes	Yes
N	9726	29012	9726	29012	9042	24516
adj. R2	0.299	0.281	0.415	0.365	0.225	0.419

*, **, and *** denote statistical significance at the 10, 5, and 1% levels, respectively. t-values are in parentheses.

#### 4.4.2 Instrumental variables

We chose the instrumental variable method to test the results to overcome the endogeneity problem caused by omitted variables. First, we choose the annual average industry index of CEO career focus as the instrumental variable, which is related to the independent variable of CEO career focus and has exogenous characteristics. The core of the instrumental variable method is to use the two-stage least square method to estimate. In the first stage, the annual average industry index of CEO career attention and CEO career attention are regression, and the estimated value of the instrumental variable is obtained. In the second stage, the estimated average annual CEO career focus index was used to replace the original CEO career focus, and the least square regression was carried out. To eliminate endogenous problems. As shown in columns 3 and 4 of Table 5, the relationship between CEO career focus and ESG performance is still significantly negatively correlated. Therefore, our primary regression results are robust after excluding potential unobservable heterogeneity.

#### 4.4.3 ESG performance lagged by one period

We rerun the regression analysis using the lagged ESG performance as the explanatory variable. The results in columns 5 and 6 of Table 5 show that the results after re-running the regression analysis are consistent with the baseline regression results in the direction and significance of key coefficients, and the results still show a significant negative correlation between CEO career focus and enterprise ESG performance. This finding further reinforces the robustness of the baseline regression results. This indicates that even when lagged ESG performance is introduced as an explanatory variable, the negative correlation between CEO career focus and ESG performance still exists and is statistically significant even when lagged ESG performance is introduced as an explanatory variable. This means that assumption 1b holds even after accounting for the temporal dynamics of ESG performance.

### 4.5 Further tests

#### 4.5.1 CEO shareholding

The results in Table 6 show that in the samples with CEO shareholding, CEO occupational attention has no significant impact on ESG Bloomberg performance, but has a significant impact on ESG Huazheng performance at a 1% significance level (ESG Bloomberg, ESG Huazheng; α = -0.956, t = -0.87; α = -0.596***, t = -5.23). In the sample without CEO ownership, CEO career focus has a significant negative impact on ESG Bloomberg performance at the 5% significance level. Has a significant negative impact on ESG Huazheng performance at the significance level of 1% (ESG Bloomberg, ESG Huazheng; α = -1.526**, t = -2.03; α = -0.326***, t = -6.24). By comparison, it is found that CEO stock ownership intensifies the inhibitory effect of CEO occupational focus on corporate ESG performance, especially in the case of ESG Huazheng performances. CEO political affiliation.

**Table 6:** Moderating effect of CEO shareholding.

	CEO owns shares		CEO owns no shares	
	ESG_Bloomberg	ESG_Huazheng	ESG_Bloomberg	ESG_Huazheng
Concern	-0.956	-0.596***	-1.526**	-0.362***
	(-0.87)	(-5.23)	(-2.03)	(-6.24)
Size	2.023***	0.452***	2.025**	0.311**
	(20.45)	(42.36)	(21.36)	(32.36)
Age	0.195***	0.006***	0.095**	0.005
	(6.81)	(5.85)	(3.62)	(1.95)
Lev	-1.926***	-0.632***	-3.416***	-0.786***
	(-2.94)	(-10.29)	(-5.95)	(-15.23)
Roe	1.136***	0.688***	0.546	0.499***
	(2.85)	(9.94)	(1.98)	(8.43)
TobinQ	-0.264***	0.056***	0.073	0.008**
	(-4.52)	(8.13)	(0.81)	(2.36)
Top1	1.945**	0.275***	1.023***	0.495***
	(2.81)	(4.36)	(1.96)	(6.31)
Indepen	3.362***	0.469***	0.885	0.195
	(5.03)	(2.95)	(0.75)	(1.758)
Ms	0.956	0.052	-4.236***	0.336
	(0.85)	(0.95)	(-3.26)	(0.98)
CEO_dual	-0.845***	-0.136***	-1.956***	-0.162***
	(-4.23)	(-6.45)	(-5.85)	(-6.82)
CEO_edu	0.956***	0.012***	0.166	0.055**
	(4.85)	(6.85)	(0.84)	(2.75)
CEO_tenure	0.175	0.056***	0.462*	0.093***
	(0.96)	(2.95)	(1.98)	(6.23)
CEO_overseas	0.856**	0.042	0.136	-0.016*
	(2.36)	(1.95)	(0.75)	(-0.74)
CEO_gender	-0.384	0.026	-0.362	-0.036
	(-1.92)	(0.98)	(-0.95)	(-1.52)
_cons	-41.452***	-3.625***	-36.032***	-2.225***
	(-12.63)	(-11.03)	(-12.42)	(-7.96)
Year	Yes	Yes	Yes	Yes
Ind	Yes	Yes	Yes	Yes
N	4962	17549	4859	13263
adj. R2	0.342	0.416	0.262	0.495

*, **, and *** denote statistical significance at the 10, 5, and 1% levels, respectively. t-values are in parentheses.

#### 4.5.2 CEO political affiliation

In the sample with CEO political affiliation, we observed that CEO professional attention has no significant effect on ESG Bloomberg performance, and has a significant negative effect on ESG Huazheng performance at a 1% significance level (ESG Bloomberg, ESG Huazheng; α = -0.859, t = -0.62; α = -0.295***, t = -3.01), in the sample without CEO political affiliation, CEO occupational attention has a significant negative impact on ESG Bloomberg performance at the significance level of 10%. Has a significant negative impact on ESG Huazheng performance at the significance level of 1% (ESG Bloomberg, ESG Huazheng; α = -1.263*, t = -1.958; α = -0.406***, t = -7.32). These results suggest that the political affiliation of the CEO has an impact on the relationship between the CEO’s career concerns and firm ESG performance. Table 7 showed the moderating effects of CEO political affiliation. Specifically, the political affiliation of a CEO exacerbates the use of a CEO’s career concerns to inhibit the firm’s ESG performance, especially when considering ESG Huazheng performance.

**Table 7:** Moderating effects of CEO political affiliation.

	CEO has political affiliations		CEO has no political affiliations	
	ESG_Bloomberg	ESG_Huazheng	ESG_Bloomberg	ESG_Huazheng
Concern	-0.859	-0.295***	-1.263*	-0.406***
	(-0.62)	(-3.01)	(-1.958)	(-7.32)
Size	1.859**	0.385***	2.336**	0.335***
	(11.36)	(29.25)	(28.03)	(48.21)
Age	0.096*	0.069***	0.195*	0.002**
	(2.98)	(5.02)	(7.16)	(2.98)
Lev	-0.845	-0.569***	-2.845***	-0.799**
	(-1.56)	(-7.13)	(-5.99)	(-18.26)
Roe	0.225	0.645***	0.816*	0.584***
	(0.43)	(5.78)	(2.62)	(10.42)
TobinQ	-0.194	0.056***	-0.081**	0.093***
	(-1.35)	(6.95)	(-2.95)	(5.95)
Top1	0.956	0.563***	1.416*	0.301***
	(1.75)	(5.03)	(2.95)	(6.85)
Indepen	3.595***	0.321***	1.702**	0.094
	(2.62)	(3.49)	(2.88)	(1.65)
Ms	3.045**	0.055	-1.846**	0.185**
	(2.98)	(0.65)	(-1.63)	(2.03)
CEO_dual	-0.733**	-0.002	-1.956***	-0.163***
	(-2.75)	(-0.46)	(-4.23)	(-9.84)
CEO_edu	0.533*	0.085***	0.498**	0.058***
	(2.98)	(4.03)	(4.13)	(5.06)
CEO_tenure	0.623***	0.044*	0.199	0.059***
	(3.16)	(1.85)	(1.41)	(5.75)
CEO_overseas	0.695	0.062	0.598**	0.006
	(1.85)	(0.84)	(1.42)	(0.92)
CEO_gender	-1.023***	-0.096**	0.195	0.036
	(-2.99)	(-2.42)	(0.57)	(0.45)
_cons	-27.452***	-3.895***	-39.625***	-3.045***
	(-5.44)	(-8.03)	(-12.61)	(-10.22)
Year	Yes	Yes	Yes	Yes
Ind	Yes	Yes	Yes	Yes
N	2852	8012	6945	21526
adj. R2	0.332	0.416	0.323	0.246

*, **, and *** denote statistical significance at the 10, 5, and 1% levels, respectively. t-values are in parentheses.

## 5 Conclusion

Starting from the perspective of high-level theory and career focus, this article uses multiple regression analysis methods to explore the relationship between CEO career focus and corporate environmental, social, and corporate governance (ESG) performance, using data from Chinese A-share companies listed in Shanghai and Shenzhen from 2011 to 2020. This study fully considers the impact of various potential factors on the relationship between CEO career attention and corporate environmental, social, and corporate governance (ESG) performance, including changes in CEO personal characteristics and market environment. By using long-term span data to capture recent trends, we can better understand the dynamic relationship between CEO career focus and ESG performance. The detailed conclusions were as follows:

1. A CEO who is highly concerned about career development can suppress a company’s environmental, social, and corporate governance (ESG) performance. This perspective provides us with a new perspective on the impact of CEO behavior on ESG performance, and compensates for the shortcomings of previous research on the relationship between CEO behavior and ESG.
2. The study also found that the CEO’s shareholding ratio as a moderating factor exacerbates the inhibitory effect of CEO professional attention on ESG performance of the company. For investors, this discovery also requires a more comprehensive consideration of the CEO’s equity structure in investment decisions.
3. CEO political affiliation also exacerbates the negative correlation between CEO professional attention and corporate ESG performance. The research conclusion can help governments and regulatory agencies take certain policy measures to monitor and regulate the political activities of CEOs, ensuring that their political connections do not improperly affect the ESG performance of enterprises.

## 6 Limitations and future research directions

There are still limitations to this study as follows:

1. different studies vary from person to person, with significant differences in the selected samples and different scoring criteria for selecting ESG, which may lead to differences in conclusions.
2. under uncertain conditions, differences in ESG scores, market stock prices, and other factors may affect the company’s liquidity due to investment returns from investors; (iii)other factors may affect ESG ratings.

Given some limitations of current research, we have proposed the following further research directions:

1. It is recommended to search for more diverse data sources and use different analytical methods. For example, raw data can be used to reduce dependence on existing indicators, which will help address potential endogeneity issues and missing variable bias in multiple regression analysis.
2. We believe that methods such as panel data analysis and causal reasoning can be explored to more fully validate our findings while addressing the challenges of dynamic and nonlinear relationships.
3. In order to more accurately measure the level of CEO professional attention, we hope to develop more objective indicators in the future. In addition to indirect measurements based on age, multidimensional indicators such as career path, leadership experience, career planning, goal setting, personal motivation, and autonomy can also be considered. This alleviates the limitations of current measurement methods and provides more comprehensive insights. These new research directions will help us gain a more comprehensive understanding of the complex relationship between CEO career focus and corporate performance.

## Supporting information

S1 File (ZIP)
